# Additive Manufacturing of a Microbial Fuel Cell—A detailed study

**DOI:** 10.1038/srep17373

**Published:** 2015-11-27

**Authors:** Flaviana Calignano, Tonia Tommasi, Diego Manfredi, Alessandro Chiolerio

**Affiliations:** 1 Center for Space Human Robotics, Istituto Italiano di Tecnologia, Corso Trento, 21, 10129 Turin, Italy

## Abstract

In contemporary society we observe an everlasting permeation of electron devices, smartphones, portable computing tools. The tiniest living organisms on Earth could become the key to address this challenge: energy generation by bacterial processes from renewable stocks/waste through devices such as microbial fuel cells (MFCs). However, the application of this solution was limited by a moderately low efficiency. We explored the limits, if any, of additive manufacturing (AM) technology to fabricate a fully AM-based powering device, exploiting low density, open porosities able to host the microbes, systems easy to fuel continuously and to run safely. We obtained an optimal energy recovery close to 3 kWh m^−3^ per day that can power sensors and low-power appliances, allowing data processing and transmission from remote/harsh environments.

In modern society it is possible to observe an everlasting permeation of electron devices and appliances, smartphones, sensor nodes and portable computing tools. Koomey’s law guarantees that the computing efficiency doubles every 1.57 years, trespassing the growth efficiency of rechargeable energy sources, which is substantially constant^1^. Many researchers see the renewable bioenergy as one of the ways to deal with the modern requirements of energy. This has led, in recent years, to focus attention on the generation of energy by means of microorganisms. It is known that microorganisms can produce fuels, such as ethanol, butanol, methane and hydrogen. In recent years, an innovative system for direct electricity production from renewable biomass, biomass-derived waste, wastewater, soil, active sludge and natural water environment and sediments was developed under the name of Microbial Fuel Cell (MFC); MFCs may enable metal contaminant removal from polluted sites. By utilizing microbial metabolism an MFC produces an electrical current from the degradation of organic/inorganic matter^2^,^3^: microorganisms convert the energy stored in biodegradable organic and inorganic compounds to electrical energy. Microbes release electrons to the anodes, and they are transferred through the load to the cathode, where they combine with protons and electron acceptors, driving a reduction reaction. Different applications were proposed for this technology, such as wastewater treatment, biosensors, energy recovery in remote areas/harsh environments/space and robotic applications^4^,^5^,^6^,^7^. The MFC anodic material is crucial toward high performance and scalability (either up or down), according to the final use. In fact the anode works not only as a conductor, but also as a bacteria carrier, hence surface roughness, good biocompatibility, efficient electron transfer between bacteria and electrode surface, are essential keys to promote biocatalytic activity^7^. Recently, modification of the anode using different materials, which can be expected to facilitate bacterial adhesion and electron transfer to the anode surface, has been a successful approach for improving power production in MFCs. These modification methods include surface treatments with physical or chemical methods, addition of highly conductive or electroactive coatings, and use of metal-graphite composite electrodes^8^. Some current studies have developed sustainable and low cost anodes from natural materials for MFCs^9^,^10^. However, these anodes show some limitations: a structure with variable size of the pores, the smaller ones often clogged because of biofilm propagation^9^, causing in the long run impractical operability for obstruction and consequently poor nutrients diffusion. An alternative to traditional processes that allow obtaining an ideal anode electrode with a high surface area, high conductivity, biocompatibility, chemical stability, and three-dimensional (3D) macroporous structure, could be the use of Additive Manufacturing (AM) techniques.

The parts obtained with this kind of processes could exhibit a tailored, interconnected porosity and surface features which should encourage the attachment of bacteria. The AM approach has seen tremendous growth in the last decade owing to convergent efforts in engineering, biology and material science. Many important application domains have benefited: from medical implants to drug delivery, from tissue engineering to harsh environment components, from consumer/leisure to building industry^11^,^12^. Recently, some researchers have begun to use AM technologies to expand the range of possible MFCs geometries and improve the simplicity of fabrication: for example to create compact external structure without fixtures, like screws, clips or clamps, using three different thermoplastics (medical-grade biocompatible polycarbonate (PC-ISO), acrylonitrile butadiene styrene (ABS), ceramic-filled photo curable resin (RC25)) manufactured by Stereolithography and Fused Deposition Modelling (FDM)^13^,^14^; in another case to produce an ion exchange membrane in Tangoplus polymer and natural rubber latex using a PolyJet 3D printing^15^. Researchers at DOE’s National Energy Technology Laboratory employed AM techniques to engineer more ideal cathode configurations in Solid oxide fuel cells (SOFCs)^16^. Their investigations of the internal SOFC structure resulted in complete and detailed 3-D maps of active components, and they are applying that knowledge to determine exactly where the electrocatalyst should be placed for optimal performance. By controlling the manufacturing of the structure, the networks of solid and gas interfaces can be better connected for improved efficiency. In other studies it was demonstrated how AM technologies significantly expanded the range of novel MFC architectures^17^ and electrodes^18^ in order to improve also the economic viability of existing configurations.

However the great advantage of the high degree of design freedom which AM provides to manufacture individual customized plastic and metal parts, could be used above all to improve the functionality of the system. Considering this aspect, biomimicry — the development of solutions based on biological adaptations — is one of the most exciting frontiers in science^19^,^20^. Leonardo da Vinci stated “In her (nature’s) inventions, nothing is lacking, and nothing is superfluous”^21^.

In this study, three different AM techniques were employed to produce a new type of MFCs, from the outer packaging, to the inner functional system. Selective Laser Melting (SLM) process was used to fabricate a bio-inspired lattice aluminum alloy anode, a 3D macroporous structure with low density, high specific surface area and a customized surface roughness ideal to host the microbes.

Marine water was chosen as inoculum because it is a complex high-conductivity and active ecosystem of major importance in the global carbon cycle. It contains a high number of diverse planktonic populations, where the resident population degrades the organic matter naturally present in seawater. Mixed cultures obtained from seawater inoculated into anodic chamber of MFC assemblies can be used in natural environment (e.g. seawater) as biological sensors^22^.

Aluminum is an active metal and its resistance to corrosion depends on the formation of the protective oxide film^23^. In general, it shows good levels of corrosion resistance in seawater and in marine atmosphere due to its ability to develop spontaneously a closed surface layer of electrically conductive aluminum oxide in the atmosphere and in nearly neutral waters^24^. Then spray coating technique was used to deposit bacteria on the same metallic structure, resulting simple, fast and efficient to obtain a uniform distribution. Furthermore, the outer packaging was designed and fabricated by means of Fused Deposition Modelling (FDM). The new AM MFC so obtained was then characterized being in function for five months and the results proof the feasibility of the approach and the advantages provided by AM techniques.

## Result and Discussion

### Design and fabrication of an AM MFC device

For the external structure, FDM was chosen to produce an MFC with two chambers (for the anodic electrode and for the cathodic one) that could be opened with a unique non-assembly mechanism^25^, as well as four connectors for the reference electrode, two for refilling of the MFC cell (inlet and outlet), and the other for the electrical circuit. The non-assembly mechanism guarantees an easier assembly of the cell and a better seal thanks to the use of two gaskets and three screws for closure. For the core of the MFC, the anode was fabricated using SLM, in which a high power laser is used to melt a powder feedstock to form fully dense metallic parts, fit for end-user products. The metallic anode was conceived as a cellular lattice structure^26^, trying to take benefits from bioinspiration: trabecular bones, wood, cuttlefish bones, corals, sponges, and bird beak interiors are only few examples of cellular hierarchical structures^27^. They can offer features such as high strength, low density, good mechanical energy absorption characteristics^26^,^28^ and open-channel flows for hydraulic permeation of nutrients. Furthermore, in order to obtain a good control of network formation of the bacteria on the anode, the spray-coating was utilized to deposit mixed bacterial suspension on the cellular lattice structure. The spray-coating is an AM technique that ensures an ideal bacteria film coating on a variety of surfaces with different morphologies and topographies. In spray-coating systems, a fluid (or an ink) is atomized at the nozzle by pressure or ultrasound and then conveyed toward the substrate by a gas. The spray technique is a solution we used at the beginning of the self colonization process, in order to distribute more homogenously bacteria seeds, if compared to the actual method to introduce them that goes through liquid injection in the MFC chamber. The spray technique represents a proof of concept towards feasibility and industrial application of AM-MFCs. In fact, it will be possible to develop in a viable way a spray with a selected lyophilized anaerobic population in O_2_-free atmosphere.

**Figure 1:**
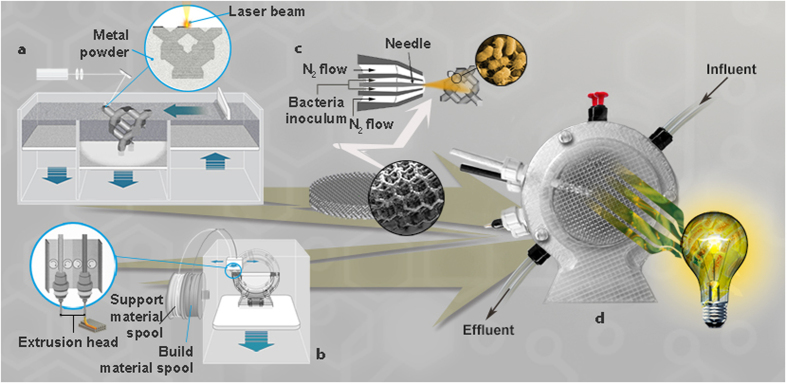
The three additive manufacturing techniques used to fabricate innovative Microbial Fuel Cells (MFCs). (**A**) In Selective Laser Melting (SLM) technology the 3D component to be fabricated is sliced into layers with a chosen thickness (30 μm) and sent to the SLM machine. The process starts by applying layer of the powder material to the building platform, and by fully melting the powders following the path corresponding to the first section of the part. The substrate platform then drops one layer thickness in the z axis before the material is recoated, and the process is repeated until the entire part is complete. (**B**) Fused Deposition Modelling (FDM) technology build parts layer-by-layer from the bottom up by heating and extruding a thermoplastic filament. The 3D printer heats the thermoplastic to a semi-liquid state and deposits it in ultra-fine beads along the extrusion path. (**C**) Anest-Iwata spray gun was used for bacteria spray-coating. Five ml of inoculum (~10% of anodic solution) was directly sprayed to the 3D anode surface of Al structure by airbrush connected to nitrogen gas, and conserved immersed at 4 °C in a liquid medium till the (**D**) MFC assembly.

**Figure 2:**
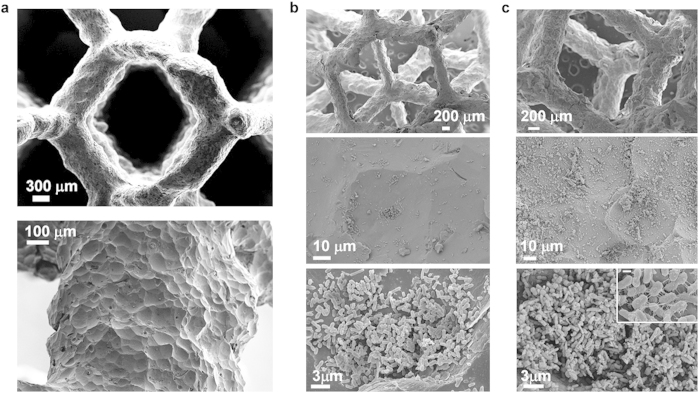
The cellular AlSi10Mg anodic structure made by SLM as appears at FESEM (Field Emission Scanning Electron Microscope). (**A**) After shot peening with ceramic microspheres: this post processing is used to improve external surface finish and to remove not consolidated powders. In this study, zirconia beads of 100–200 μm were used creating a unique rough surface characterized by niches with a mean diameter ranging from 50 μm to 100 μm, covering all the struts of the lattice structure, as can be seen at higher magnifications. (**B**) The same lattice structure after bacteria deposition using spray coating: as supposed, the bacteria primary stay inside the preferential sites (niches). (**C**) Finally the same structure after testing in MFC for 5 months; at higher magnifications it is possible to appreciate the bacteria proliferation and the network of pilum. The scale bar of the inset is 300 nm.

Figure 1 displays the three AM techniques used in this study. Starting from the knowledge of the lattice structures that can be built by SLM without deformations^29^, a cylinder shaped anode in AlSi10Mg alloy of 65 mm diameter and 15 mm height designed through the repetition of a Schwartz Diamond unit cell was fabricated by direct metal laser sintering (DMLS, the trade name of EOS GmbH for SLM). The diamond lattice structure is self-supporting: this means that it could be manufactured without support structures, which are commonly used in SLM to hold unsupported geometries in place and to prevent deformation by thermal stresses typical of this process, but they are not acceptable inside complex structures because in this case they cannot be removed. A unit cell of 4 mm with a volume fraction of 20% was chosen, being the optimum compromise between a high specific surface, a structure with good manufacturability^29^ and a high percentage of voids. The designed lattice structure has circular struts (bridging nodes), with a mean theoretical diameter of 0.85 mm. The aluminum alloy was chosen due to its high electrical conductivity, low density (2.68 g cm^−3^), good corrosion resistance and very good manufacturability by SLM^30^,^31^. All these factors give to the anode component a theoretical density of 0.54 g cm^−3^. To avoid any contamination due to the loose powder, the aluminum manufactured sample was post processed using shot peening. The surface of the parts produced by SLM could be greatly influenced by the “balling” phenomenon that occurs during laser melting causing the formation of discontinuous tracks, and by the presence of partially-bonded particles on the top surfaces^32^. Depending on the application, this surface morphology is desirable to obtain better results. For example in the biomedical field, it is well recognized that the surface roughness improves the osteointegration^33^, thanks to intercommunicating cavities that replicate the bone structure. These concavities could penetrate deep inside the implant body, creating pits and pores that are colonized by bone cells. Shot peening, having an effect on the surface, not only eliminates the partially-bonded particles but also enhances surface finishing^30^, and improves the corrosion-resistance by closing the open porosities. Furthermore, using zirconia beads it is possible to create on an aluminum structure a sort of textured surface made of niches at the microscale. This is ideal for the bacteria employed in the MFC, as shown in Fig. 2. Moreover, before the use inside the anodic chamber, it was conducted biocompatibility test on the AM electrode in plate-count agar (see Methods) in order to understand if aluminium structure was suitable to host microorganisms for their growth. The results has been confirmed that AM electrode has good affinity with bacteria (Fig. 2).

As already observed, the surface features of anodic materials are one of the deciding factors that affect bacterial attachment and charge transfer between bacteria and the electrode surface. Furthermore good nutrient diffusion is ensured without clogging, thanks to the high vacuum degree (~80% theoretical) and high electrical conductivity. Moreover, the spray coating technique, never been used for MFCs, results efficacious, easy and fast and practical from small to large scale, permitting uniform bacteria proliferation and hence, ensuring a fast start-up of MFC system.

### AM MFC performances

We designed the AM MFC system in a way similar to hydrogen fuel cells, where a cation exchange membrane (CEM) is sandwiched between the anode and cathode^34^. The architecture was chosen in order to reduce as much as possible the inter-electrode distances between anode and cathode, with the aim of reducing ohmic resistances and resulting in a significant increase of power output. The influence of electrode spacing on performance of MFCs has been shown in several works^35^,^36^,^37^,^38^. Another architectural parameter that leads to better performances is the active electrode surface area: the more active electrode area available, the more bioelectricity generation guaranteed for a given volume of the cell. Several works have investigated the use of reticulated and granular conductive carbon instead of flat electrodes, but sometimes their heavy and relatively low porosities slow metabolic and kinetics reactions for both biofilm and nutrients flow clogging^39^,^40^,^41^.

For this reason, the combination of these two design parameters (inter electrode distances and high surface area), together with their open channel flows permit to have a high-performance anodic materials for MFC. The AM MFC operated for more than 5 months (under external loads ranging from 10 to 820 Ω) and a continuous feeding of anodic organic substrates with an Hydraulic Retention Time (HRT) of ~6 days and demonstrated stable and reproducible performance. Traditional waste water treatments based on aerobic technologies are characterized by a shorter HRT, in the range 1–4 days. Nevertheless in an anodic chamber such as that of an MFC, anaerobic conditions usually require a higher HRT^42^ (15–40 days) to ensure retention of the slowly growing anaerobic microorganisms. The AM-MFC system proposed by us, thanks to the AM-3D support that enhances the attachment of microorganisms, permits to work with liquid passing through the chamber at a faster rate around 6 days.

**Figure 3:**
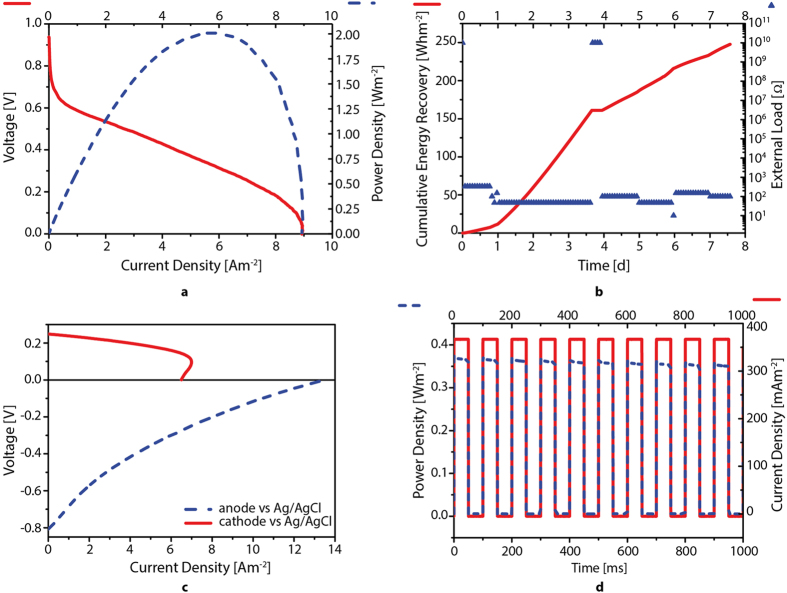
Electrical characterization of AM manufactured MFCs. From top to bottom, from left to right: (**A**) polarization curve (Voltage vs current density curve) and power density curve of MFC; (**B**) total energy recovered in a week by MFC under external loads (from 820 to 10 Ω, open circuit condition 10^10^ Ω); (**C**) polarization curve of both semicells (anode and cathode); (**D**) pulsed power density, at fixed currents of either 412 ± 1 mA m^−2^ or 0 mA m^−2^.

Representative performances under different electrochemical techniques are reported in Fig. 3. Figure 3A shows the polarization and power density curves of the complete MFC cells. Polarization curve shows that when no current flows through the system, the voltage is at almost 1 V (Open Circuit Voltage, OCV conditions). According to the 1 mV s^−1^ decrease of voltage, current slowly increased till reaching the maximal current production (Short Current Circuit, SCC) of about 9 A m^−2^. The maximum power point (MPP) reached by polarization curve was 2 W m^−2^ at 0.3 V and 6 A m^−2^. According to the anodic volume of MFC and the AM-structure with 80% of vacuum degree, the power density obtained was 148.8 mW L^−1^ (Table 1). Moreover, from polarization curve, we can discern three different zones, where prevails different resistances,

- Activation resistance (*R*_*a*_), also called charge-transfer resistance, is visible in the first part of a polarization curve at low currents, thus near to open circuit voltage (OCV). It depends on catalysts, electrochemical mediators, biofilm, microbial species and their metabolisms, and operative conditions such as temperature and pH. It derives from the slowness and irreversibility of the reactions taking place on the surface of the electrodes^43^,^44^.
- Ohmic resistance (*R*_*Ω*_) is visible in the linear segment at intermediate currents in the polarization curve. It is mainly caused by ionic resistances of electrolyte, membrane and biofilm, and by electronic resistances of electrodes, current collectors and electrical connections^43^,^44^.
- Concentration or mass-transfer limited polarization resistance *R*_*c.*_is visible in the high current part of a polarization curve, thus near to short circuit current (SCC). *R*_*c*_ mainly depends on the rate of electrons and protons diffusion, required to sustain the generation of current. Thus, this overpotential is prevalent at high current densities when there are more charges produced that need to be transferred from the electrolyte bulk to the electrode surface. Mass-transfer resistances are affected by geometry of the cell, electrolytes, structure of the electrodes and biofilm, metabolites and products^43^,^44^,^45^,^46^.

Therefore the internal resistance *R*_*int*_ of the MFC system can expressed in term of ohmic resistance R_Ω_ and the electrode overpotentials *R*_*a*_ and *R*_*c*._

Taking into account that in semi-cycle symmetrical polarization curve (as we can define ours curve) the MPP occurs at a point where the External Resistance *R*_*ext*_ is equal to *R*_*int*_^34^, from Ohm’s Low (*R *=* V/I*) we obtain that *R*_*ext*_ = *R*_*int*_ = 15.2 Ohm. Hence, the internal resistances *R*_*Ω*_ (10.8 Ohm) is dominant respect to the other two resistances (*R*_*a*_and *R*_*c*_) that are in the order of 5 Ohm.

Beyond polarization analysis that were performed periodically to check the electrochemical behaviour, MFCs were continuously submitted to different external loads in order to evaluate the real energy output, under fluctuations (Fig. 3B). Changes in the resistances help to evaluate how the system responds to different external stress and energy request, from electrical point of view, permitting to evaluate that the system responds well at different energy request, even at 10 Ohm resistance where 4 W/m^2^ has been recorded. Taking into account the syringe pump used, the maximal energy request is 65 J (18 Wh) per day. In average, the daily energy produced by our MFC is 402 J (112 Wh). The net energy balance is positive. Anyway, in order to decrease as much as possible the energy expenditure, that depends also from other energy cost items, it will be convenient to consider a gravitational fall of water inside the MFC systems. In fact using an up-flow system, organic water is fed from above the fuel cell, and by gravity pushed down and around, and thus pumping and mixing become unnecessary, with a positive outcome on the overall energy efficiency of the device. In this case, the energy and continuous power values obtained under loads by the MFC of more than 2.5 W m^−2^ (200 mW L^−1^ (W m^−3^)) might be referred to a net energy recovery from the MFC system. According to the energy recovered in one week, we obtained more than 18 kWh m^−3^ anodes (250 Wh m^−2^). In addition, in order to increase the overall system voltage, MFC can be stacked as in the batteries, to increase the overall system voltage. The influent COD (Chemical Oxygen Demand) of the solution continuously fed by syringe pump was 10.8 ± 0.3 g/L and the output was in the range 0.6–3.2 g/L, including some fluctuations according to the load impedance applied. The maximum COD removal obtained was 93 ± 2% obtained working at low resistances^47^ and (100 Ohm). In average, COD removal was in the range 70–95%.

Nevertheless, owing to the superficial nature of the phenomena, it is important to maintain, in the scale-up design, the same electrodes surface over liquid volume, i.e. m^2^/m^3^. Scale-up issue is certainly an aspect that should be carefully taken into account for industrial applications, considering the optimized design of devices and the opportunity to obtain the energy/voltage output required and water treatment with a series of smaller MFCs^48^,^49^,^50^. Moreover, the system working with real wastewater, according to the nature of organic substrates, should be integrated with a chemical/physical pretreatment (before the inlet toward MFC) necessary to remove large solids and other undesirable substances from the wastewater and make complex organic substrates easily metabolized by microorganisms and therefore a more efficient electron release. This pretreatment step is very common in anaerobic digester plants and aerobic treatment systems and helps to prevent the biological block of depuration, even if some decrease of electrical performances could happen.

Since we independently monitored also the anode and cathode potential, we were able to know their contributions respect to total cell. Figure 3C shows the voltage drop in both anodic and cathodic chambers due to the imposed potential at 1 mV/s of scan rate obtained by linear sweep voltammetry in the three electrode configuration. Ag/AgCl electrode was used as working reference electrode both in anodic and cathodic chamber measurements, and current at a working electrode (respectively anode and cathode) was measured. In anodic chamber, at each potential the current output was always bigger than the cathodic one. Moreover, by confronting the maximum power point (MPP) of anode that is 1.8 W/m^2^ (nearly 2 W/m^2^ for the complete cell) with that one of the cathode alone that is 0.96 W/m^2^ (about half the previous value), we concluded that the limiting one is the cathode. This behavior has been noticed frequently during 5-months experimental tests and it is completely different from what was measured using our standard (carbon felt), in which case the anode demonstrates the worst performances^43^. While the anodic potential remained stable at approximately −0.8 ± 0.1 V at OCV under low load resistances (under 100 Ω) the cathode exhibited a sharp drop of potential. This behavior is more evident in Fig. 3C that shows separately both anodic and cathodic potentials vs Ag/AgCl reference electrodes. Since the cathode vs Ag/AgCl showed a maximum short current circuit (SCC) point and power density (MPP) of 6.5 A m^−2^ and 0.9 W m^−2^ respectively, nearly half the anode vs Ag/AgCl performance rating 13.5 A m^−2^ and 1.8 W m^−2^, we believe that the rate limiting component of the AM MFC is the cathode reduction reaction mediated by ferricyanide.

It is probable that the potassium ferrycianide (Fe^3+^) rapidly tends to wear out reducing itself to Fe^2+^ due to the high electrons migration through the cathode. These results suggest that a long-term use of the AM MFC requires an improved cathode. Moreover, the system in order to be completely sustainable and with even higher efficiency should be based on cathode-oxidizing microorganisms that are able to conduct biological reduction^40^,^48^,^49^.

Table 1 is an overview of electrical parameters recovered by polarization techniques. Beyond characteristic points, such as SCC, MPP and OCV, it reports Ohmic resistances and the intrinsic resistance of AlSi10Mg, before and after a 5-months use as anode in MFC. Intrinsic resistance after use keeps very low values proofing good reliability of the electrode under continuous and long working intervals. The higher intrinsic resistance after use could be due to a slight corrosion and coating of biofilm on the surface, since cell membranes mostly contain non-conductive materials such as polysaccharides, lipids and peptidoglycans^50^.

Figure. 3D shows how the AM MFC system is fast and efficient to support intermittent variation of current and hence, power, allowing fast data transfer. Moreover, if the request is continuous, the system promptly responds. These high-performance features, both electrochemical and of robust design, suggest to consider this system layout as an interesting solution for remote/harsh environments, where the energy source (environmental organic matrix/waste) is present and conversely the connection to the conventional energy grid is a complicated issue.

## Conclusions

Microbial fuel cells are the more widely studied example of bioelectrochemical systems, performing the double task of wastewater treatment and electricity generation. In a typical microbial fuel cell, the anode is used to harvest electrons from wastewater while oxidising organic pollutants. The performance and cost of electrodes are the most important aspects in the design of MFCs. Although other studies have explored the use of metallic anodes^34^,^35^,^51^,^52^,^53^,^54^, the maximum power density reached was about 816 mW m^−2^ 54; in comparison the system layout here described under 47 Ohm resistance steadily generate over time more than 2500 mW m^−2^. The results of our research enlighten the advantages provided by additive manufacturing to increase the energy produced. 3D metal anodic structure goes exactly in that direction, simulating the coral skeletal structure, which has a network of channels very similar to a circulatory system, through which information are transferred and permitting to obtain an optimal energy recovery close to 3 kWh m^−3^ per day. Moreover, the structure with preferential niches is suitable for many species that create symbiotic relations: on the 3D structure also microbial populations can communicate and transfer electrons among the biofilm and the electrode network. To this aim, the spray coating resulted in a very innovative and easy tool to produce such “electrode bioconsortia” well distributed. In remote/harsh environments, such as deepsea platforms, manned space stations, on-board boats, a daily energy density as that we obtained can continuously power not only sensors and low-power appliances, but also data processing and transmission systems^55^. Of course, according to natural environment, other inoculums (e.g. rivers, lake, wastewater treatment) can be considered^56^,^57^,^58^. So far, the technology here described to realize the MFC represents undoubtedly a cool candidate for such applications.

## Methods

### Selective Laser Melting (SLM) process

The cellular lattice structure was manufactured through an EOSINT M270Xt DMLS machine using a gas atomized AlSi10Mg powder produced by EOS GmbH (Germany). Direct Metal Laser Sintering (DMLS) is the trade name of EOS GmhH to indicate Selective Laser Melting (SLM). In this machine, a 200 W Yb fiber laser system with nominal diameter of 100 μm is used in Ar atmosphere to melt selectively the powders in 30-μm-thick layers. The particles have a regular spherical shape, with a diameter ranging from 0.5 to 30 μm, and a mean size of 20 μm. At the end of the process, the building platform with the lattice structure is removed from the DMLS machine and is put into a furnace for a stress relieving treatment (2 hours at 300 °C). After this the part is detached and post processed with shot peening. After that the struts of the AlSi10Mg diamond cell unit obtained had a mean diameter of 0.68 mm. Thus the resulting density of the cellular anodic structure was 0.43 g/cm^3^, 20% less than the theoretical one. This could be ascribed to the shot peening process, which makes the struts thinner.

### Diamond lattice geometry

Diamond lattice structures are mathematically defined by triple periodic implicit functions, allowing precise control of volume fraction and unit cell size in the creation of an interconnected network. In mathematical terms, let *S* be a surface defined by (Eq. 1):


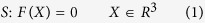


where *X* is a point of coordinates x, y and z. The surface *S* represents the border between the matter and the voids. A trigonometric polynomial has been used for the definition of the function *F(X),* which can be written as a sum of d terms as shown in Eq. 2:


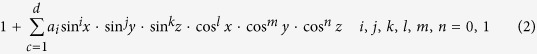


This gives rise to triply periodic level surfaces^59^: the primitive surface, the diamond surface and the gyroid surface, having interconnectivity orders respectively equal to 6, 4 and 3. The equation for a diamond surface is (Eq. 3):


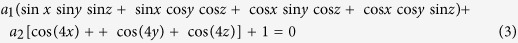


Not only the solid constituents determine the properties of cellular materials, but also the spatial configuration of voids and solid – that is, the cellular architecture^60^. Metal stochastic porous structures typically have a random distribution of open or closed voids, whereas metal periodic cellular lattice structures have uniform parts that are based on repeating unit cells. When the cell shape is fixed, the cells can be stacked in more than one way giving structures which have differing edge connectivity, and different properties^59^. The periodic channels created with this diamond unit cell were very useful to allow a fast recirculation of water inside the MFC, refreshing the live food for the bacteria. At the same time, the microstructure of the AlSi10Mg struts is extremely fine and features a controllable texture, exhibiting superior mechanical properties in comparison to an as cast aluminum alloy of a similar chemical composition.

### Biocompatibily tests on AM structure

Biocompatibity tests on the cellular structure were conducted before its use inside the anodic chamber, in order to assess the interaction between bacteria and material and to understand if it is suitable to host microorganisms for their growth. The test has been conducted in plate-count agar by including the AM-electrode inside the solid agar media (composition in g L^−1^ dissolved in mineral water: 7 C_6_H_12_O_6_, 8 C_2_H_3_NaO_2_, 10 peptone, 15 agar, 8 Na_2_HPO_4_, 5 NaH_2_PO_4_) and inoculating 10^−3^ diluted enriched sea-water culture around the AM-electrode surface and on it. Duplicate cultures were incubated at 30 °C for 48 h together with blank test (without AM). After that, we have evaluated the Colony-Forming Unit (CFU) in the agar and in particular if colonies were present on AM-electrode surface. Plate-dish samples comparison demonstrate that material has good affinity with bacteria.

### MFC design and operation

The experiments were run at room temperature (22 ± 2 °C) in continuous feeding mode, using a Syringe Pump (NE-1600 Programmable Syringe Pump, USA) with Hydraulic Retention Time (HRT) of 6 days for each MFC, feeding a synthetic substrate at pH 7 and the organic loading rate of sodium acetate and peptone of 1 and 1.25 g L^−1^day^−1^, respectively. The total anolyte volume of MFC assembly is 46.2 ml. MFCs were inoculated in the anode chamber by mixed culture coming from seawater (Arma di Taggia (IM), Italy). The mixed culture, before being inoculated in MFCs was previously enriched in batch, with a medium containing acetate, peptone, phosphate buffer and 1% of agar, in order to favourite microbial growth before inoculation in MFCs^55^. Five ml of inoculum (~10% of anodic solution) was directly sprayed to the 3-D anode surface of Al structure by airbrush (Iwata) connected to nitrogen gas, and preserved immersed at 4 °C in a liquid medium in N_2_ atmosphere, till the MFC was assembled. A carbon felt (Soft felt SIGRATHERM GFA5, SGL Carbon, Germany) was used as cathode electrode and assembled together with a graphite rod (diameter 5 mm, SGL Carbon, Germany) to ensure an effective current conduction capability. The cathodic compartment was filled by potassium ferricyanide (6.58 g L^−1^), added as oxidant compound, and a buffer solution of mineral salts Na_2_HPO_4_ (8.2 g L^−1^) and NaH_2_PO_4_ (5.2 g L^−1^) was used. Moreover, optical density (OD) analysis by UV-spectrophotometer at 600 nm was conducted in the liquid phase of anodic chamber for monitoring the planktonic population growth during the first days of experiments and occasionally for a check.

### FESEM preparative

Small portions (1 cm^3^) of anode electrodes with bacteria on the surface (after bacteria spray coating process and after testing in MFC for 5 months) were fixed adding 2.5% v/v formaldehyde in 0.1 M phosphate buffer solution and incubated at 4 °C overnight. The samples were then washed twice with 0.1 M phosphate buffer and dehydrated by successive 10 minutes incubations in 40%, 60%, 80%, 90% and 100% ethanol. The samples were then dried with a critical-point drier and coated with gold using an ion-sputter to a thickness of 10 nm. After this procedure, specimens were examined by FESEM.

## Additional Information

**How to cite this article**: Calignano, F. *et al.* Additive Manufacturing of a Microbial Fuel Cell - A detailed study. *Sci. Rep.* **5**, 17373; doi: 10.1038/srep17373 (2015).

## Figures and Tables

**Table 1:** Overview of selected properties of AM-MFC, under electrochemical analysis.

Resistance [*Ω*]			Voltage	Current	Power
Ohmic Resistance Whole AM-MFCassembly	10.6 ± 0.4	Open Circuit Voltage (OCV)	0.93 V	0 A	0 W
Anode AlSi10Mg before use	0.9225 ± 0.0005	Short Circuit Current (SCC)	0 V	32.40 mA	0 W
				701.94 mA L^−1^	
				8.93 A m^−2^	
Anode AlSi10Mg after use	1.939 ± 0.003	Maximum Power Point (MPP)	0.32 V	21.24 mA	6.87 mW
				460.00 mA L^−1^	148.82 mW L^−1^
				5.85 A m^−2^	2.01 W m^−2^

Resistance anode AlSi10Mg before use and after 5 months running test.
